# Virtual reality-based neurological examination teaching tool(VRNET) versus standardized patient in teaching neurological examinations for the medical students: a randomized, single-blind study

**DOI:** 10.1186/s12909-021-02920-4

**Published:** 2021-09-15

**Authors:** Sang Gil Han, Young Dae Kim, Tae Young Kong, Junho Cho

**Affiliations:** 1 Department of Emergency Medicine, Yonsei University College of Medicine, 50 Yonsei-ro, Seodaemun-gu, Seoul, 03722 Republic of Korea; 2 Department of Neurology, Yonsei University College of Medicine, 50 Yonsei-ro, Seodaemun-gu, Seoul, 03722 Republic of Korea

**Keywords:** Virtual reality, Neurological examination, Medical education, Standardized patient

## Abstract

**Background:**

The conventional methods for teaching neurological examination with real patients to medical students have some limitations if the patient with the symptom or disease is not available. Therefore, we developed a Virtual Reality-based Neurological Examination Teaching Tool (VRNET) and evaluated its usefulness in in teaching neurological examinations for the medical students.

**Methods:**

In this prospective, randomized, single-blind study, we recruited 98 medical students and divided them into two groups: 1) A standardized patient(SP) group that received the clinical performance examination utilizing standard patients complaining of dizziness was provided neurological findings using conventional method such as verbal description, photographs, and video clips; 2) A SP with VRNET group that was provided the neurological findings using the newly developed tool. Among the 98 students, 3 did not agree to participate, and 95 were enrolled in this study. The SP group comprised 39 students and the SP with VRNET group had 56 students.

**Results:**

There were no statistical differences in VRNET’s realness and student satisfaction between the SP and SP with VRNET groups. However, a statistically significant difference was found in the Neurologic Physical Exam (NPE) score (*p* = 0.043); the SP with VRNET group had higher NPE scores (3.81 ± 0.92) than the SP group (3.40 ± 1.01).

**Conclusions:**

VRNET is useful in teaching senior (graduating) medical students with SP with a neurologic problem.

**Supplementary Information:**

The online version contains supplementary material available at 10.1186/s12909-021-02920-4.

## Background

Virtual reality(VR) can be defined as “a computer-generated simulation of the real or imagined environment or world.” [1] VR is best described as a concept of advanced human–computer interaction. VR enables humans to directly interact with computers in computer-generated environments that simulate our physical world [2]. Standardized patient(SP) is a “patient-actor” who has been trained to consistently portray a specific patient role, outlined by a script devised by topic content experts (eg, the course coordinators) [3].

The application of VR and SP in medical education invokes elements of at least two pedagogical frameworks. The first one is social learning theories that learner’s experience is partially shaped by the context in which learning takes place. The second is a transformative learning theory that critical reflection can be used to challenge the learner’s beliefs and assumptions when medical education using SP or VR coupled with feedback and reflection [4].

Both VR and SP have emerged to educate medical trainees because the classical concept of “learning by doing” has become less acceptable [2]. Providing sufficient opportunities for medical students to examine real patient experiences through clinical clerkship can be difficult because the greater emphasis is often placed on patient safety and rights, fewer opportunities students can have, even though such experiences form an important part of medical students’ training. Furthermore, if the patient with the symptom or disease considered necessary for education does not visit the hospital timely during the student’s education period, then that student may not have the opportunity to gain experience about that particular symptom or disease.

Under these constraints, VR could be an excellent alternative for educating medical students despite some drawbacks. VR is not suitable for simple task training such as abdominal palpation or cannulation. VR characters are not yet suitable for teaching how to deliver breaking bad news because facial expressions are, at present, best covered by a human rather than a virtual patient. Nevertheless, VR has its advantages in medical education. VR makes students accessing clinical experiences simply. VR scenarios can be made whatever it is if needed for education and repeatable that allows learners to make mistakes safely and then learn through deliberate practice to improve performance. Any virtual scenario could also be objective and standardized, ensuring consistent quality and adherence to the latest protocols, so students can practice the latest protocols [5].

For medical students, the neurological examination is a foundational clinical skill in which they are expected to gain ability and competence before graduation. For neurological examinations, doctor–patient interactions are more important compared to other clinical skills; therefore, neurological examination training for medical students is typically conducted using standardized patients (SPs) rather than simple task trainers or high-fidelity manikin simulators [6–8]. In many cases, medical students learn about abnormal neurologic findings, especially non-voluntary reactions such as light reflex, nystagmus, corneal reflex, doll’s eye, and facial palsy, through verbal descriptions, photos, and video clips. Indeed, the presentations of abnormal findings through words, pictures, or video clips differ from how they emerge in actual clinical situations. Furthermore, such educational visual materials, such as photos and video clips, are not always readily available for use, and medical students may not be able to improve their clinical diagnostic ability to personally determine whether a given set of findings is normal or abnormal.

Virtual reality (VR) medical education can overcome some of the limitations of conventional educational methods by providing abnormal neurological findings that SPs or manikins cannot directly express. Moreover, basic VR-based medical education tools can allow medical students to perform infinite repetitive training, regardless of time and place [9].

Therefore, we developed a virtual reality-based neurologic examination teaching tool (VRNET) that can indicate abnormal neurologic findings that SPs cannot express. This study was aimed to evaluate the usefulness of the VRNET in teaching senior (graduating) medical students with SP with a neurologic problem.

## Methods

### Virtual reality software setup

We developed VRNET, a VR program that performs neurological tests using Oculus Rift, a popular VR headset developed by Oculus VR (a division of Facebook) (Oculus VR, SF, USA), between September 2018 and February 2019. We commissioned a company named FNI (Korea, http://fnikorea.com/), a VR content developer, for software development. The following tools were used in the software development: 3Ds Max (2014 Ver., AUTODESK, USA) for background and characters and Photoshop (CS6, Adobe, USA) for texture and user interface (UI). The graphic engine utilized was Unity (Version 5.5.6f2, Unity Technologies, USA), and the development kit was provided by Oculus VR. The computer hardware specification used for connecting the Oculus Rift was an Asus ROG GK702V (64 bit) computer (Intel Core i7-7700HQ @ 2.80 GHz, RAM DDR4 16 GB 2400 MHz, NVIDIA GeForce GTX 1060, SSD 256 GB Samsung MZNLN256HMHQ-000H7, HDD 1 TB ST1000LM035-1RK172). This study utilized the Microsoft Windows 10 (64 bit) operating system.

**Fig. 1:**
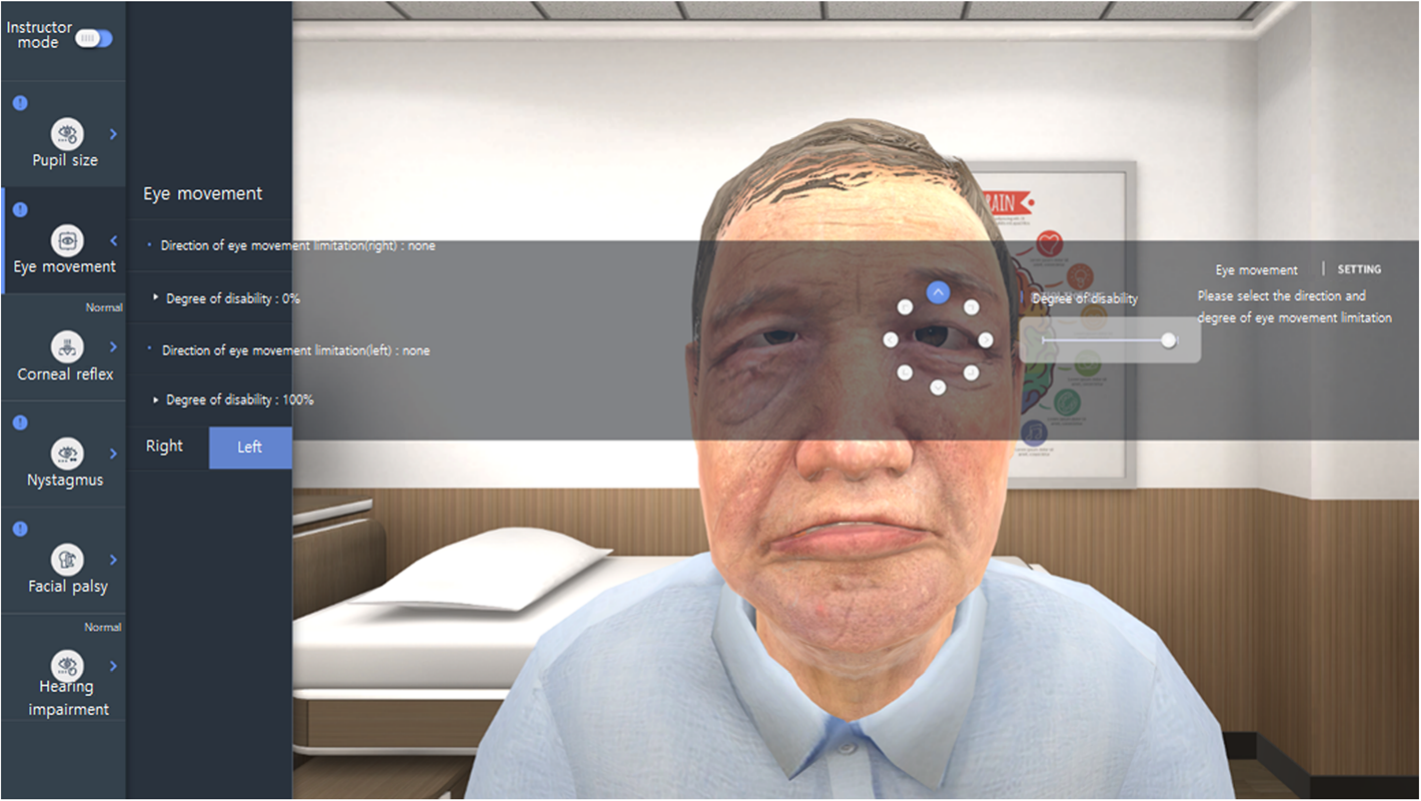
Instructor mode of VRNET. The instructor can select pupil light reflex, extraocular movement (EOM), corneal reflex, nystagmus, facial palsy and sensory change, and hearing to adjust abnormal symptoms. The figure depicts the setting of the direction and severity of the left eye EOM disorder. (This figure is a screenshot of VRNET and the person in this figure created a virtual patient)

In the classroom setting, the VR enables the instructor to set the severity or type of involuntary neurological findings, such as pupil size, pupil light reflex, corneal reflex, nystagmus, eye movement, ptosis, facial palsy (forehead wrinkle, nasolabial wrinkle, and lip movement), and hearing impairment, which SPs cannot express. If the instructor wants to change the pre-set neurological findings, then he or she can switch the mode to “instructor mode,” which enables him or her to set the severity of the type of neurological findings, such as pupil size, pupil light reflex, extraocular movement limitation, corneal reflex, nystagmus (spontaneous nystagmus only), facial palsy, and hearing impairment, by clicking the button in the top left of the computer screen. All required settings can be adjusted using the options on the left and right sides of the screen; the degree of disability for abnormal neurological findings, such as pupil size, pupil light reflex, extraocular movement limitation, nystagmus, etc., can also be set if necessary (Fig. 1).

**Fig. 2:**
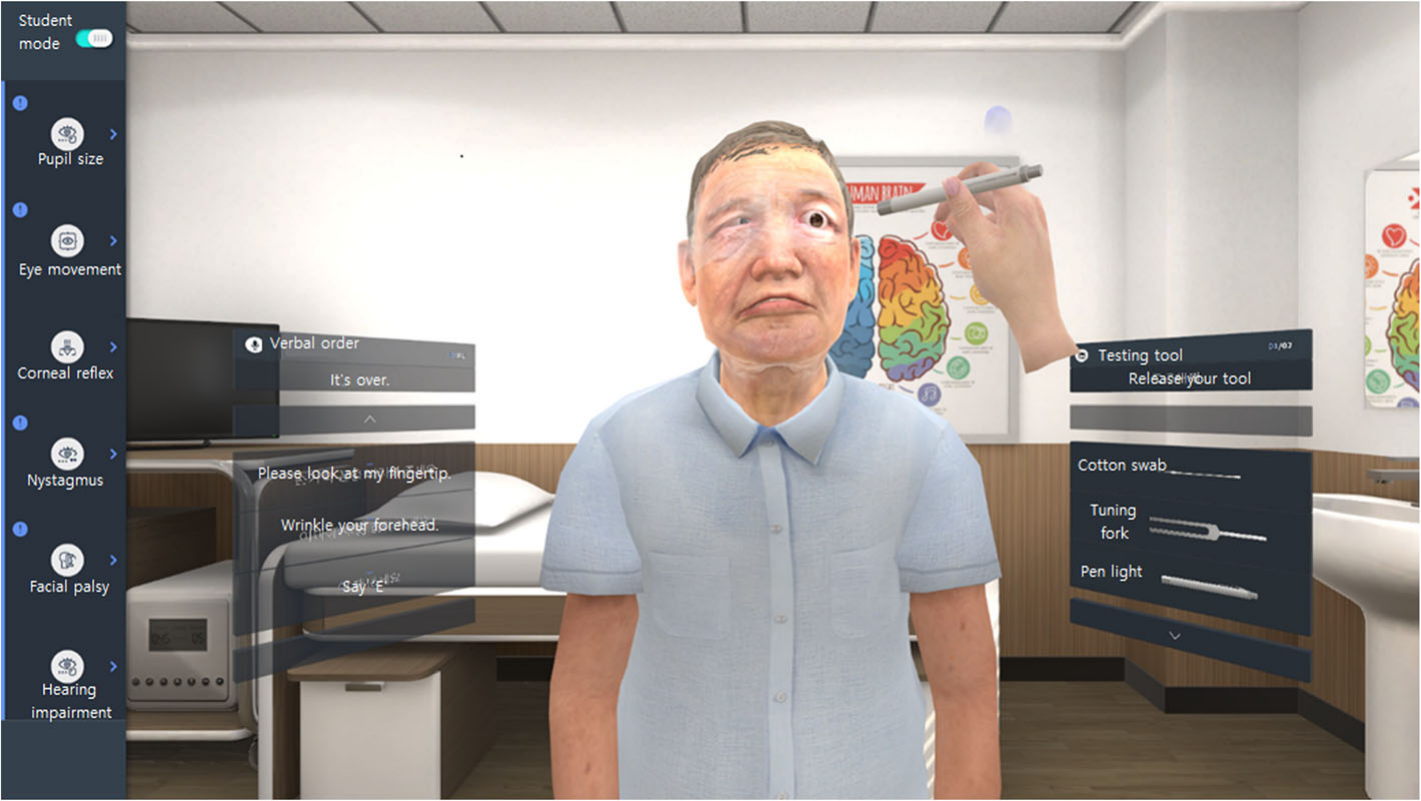
Student mode of VRNET. The student selects a voice indicator from the box to the left of the patient in order to instruct the patient and selects a neurological test tool, such as a cotton swab, a tuning fork, or a penlight, from the right box. The figure shows a pupil light reflex examination of a patient with an abnormal enlarged left pupil. (This figure is a screenshot of VRNET and the person in this figure created a virtual patient)

**Fig. 3:**
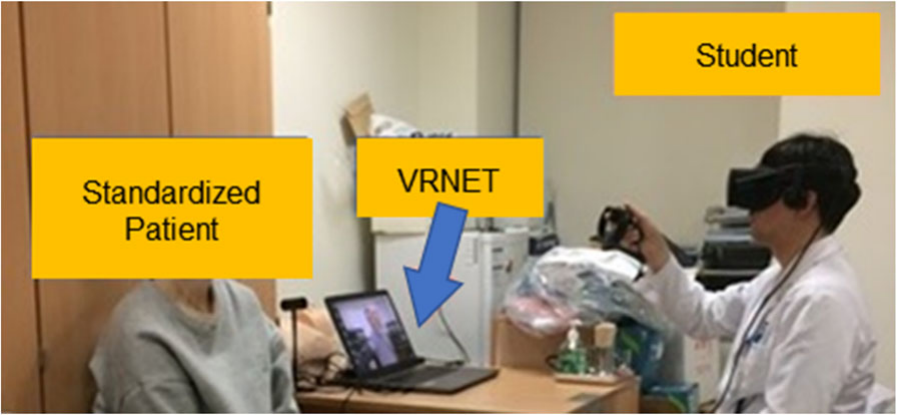
Actual training scene. The student is practicing using VRNET next to a standardized patient(this photo was taken with the prior consent of the student and standardized patient)

To perform a neurologic examination on a virtual patient, students wearing oculi select a verbal order on the left side of the screen and then select the neurologic examination tool on the right side of the screen (Figs. 2 and 3). Verbal orders consist of two parts: the questions and the directives, which are both necessary for neurological testing. The questions and directives are “Can you hear?”, “Can you feel this touch on your cheek?” “Please look at my fingertip,” “Please look at the right side,” and “Wrinkle your forehead.” VRNET offers five tools for neurologic examination. A cotton swab is used to check for a corneal reflex. A tuning fork or finger flick can be used to conduct hearing tests. The pupil light reflex is inspected using a penlight and a smartphone flash.

### Standardized patient

Two female SPs were presented during the study period. One was a 38-year-old and the other was a 45-year-old. They are a professional theater actress and have been serving as an SP for more than 5 years at our Clinical Skills Center. For this study, the training coordinator trained the scenario 2 h in advance.

### Assessment of VRNET’s usefulness

**Fig. 4:**
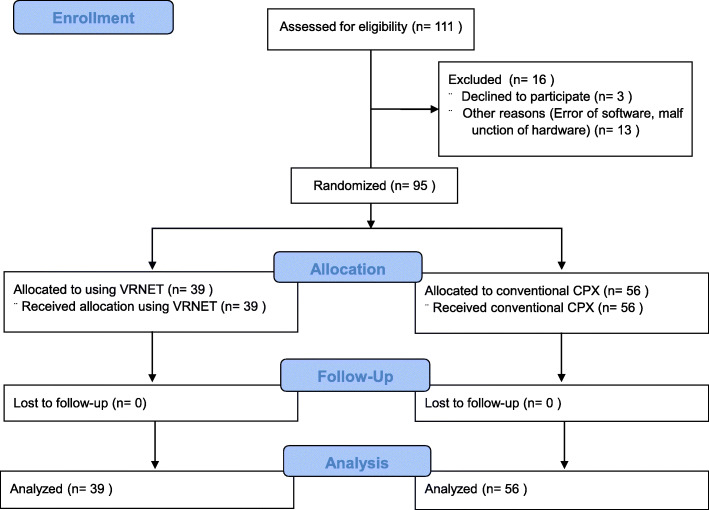
Flow chart of the study protocol

In the March–July 2019 period, a prospective randomized single-blind study was conducted for senior (fourth year) medical students during their emergency medicine clerkship. The allocation was done by tossing a coin just before the start of class. Among the 111 students who attended the emergency medicine clerkship, 13 did not participate in the study due to a compatibility issue between the VRNET computer program and the Oculus Rift. Among 98 trainees, 95 trainees were enrolled in this study (excluding 3 trainees who did not agree to participate in the study) (Fig. 4).

This study protocol was reviewed and approved by the Institutional Review Board (IRB No. 2018–1432-001), and was registered as a clinical trial (NCT03653221, Registered 31 August 2018, https://clinicaltrials.gov/ct2/show/record/NCT03653221).

During a two-week clerkship, students received the Clinical Performance Examination (CPX) training, which utilized SPs who complained of abdominal pain, chest pain, dyspnea, or dizziness. CPX training allows for the examination of one case for 30 min. One student takes 10 min to complete the following process with an SP: examine the SP, administer a questionnaire, and conduct physical and neurological examinations as required. When the SP leaves, the student explains the suspected disease and the diagnosis plan to the professor, who then provides feedback.

VRNET was used to examine SPs who complained of dizziness requiring neurological examination. The CPX case of the patient with dizziness was conducted on the second day of the emergency medicine clerkship. When a control group without VRNET examined the SP complaining of dizziness, the neurological finding was provided by conventional methods, that is, through verbal description, photos, and video clips. Meanwhile, the experimental group utilized VRNET to conduct the neurological examination. Immediately after the examination, all students completed a five-point scale indicating their realness and satisfaction with their care experiences with SPs who complained of dizziness.

On the last day of the emergency medicine clerkship, all the students performed the CPX again with an SP complaining of dizziness in the CPX room, which is capable of recording their hands-on neurological examinations. All the processes performed were recorded. A researcher who did not participate in the training measured the Neurologic Physical Exam (NPE) score (0.0–6.0) by reviewing the recorded video of medical students’ CPX examination. A composite Neurologic Physical Exam (NPE) score was developed by a neurologist and educators from the Margaret and Ian Smith Clinical Skills Center. The scoring system took into account the cranial nerve, motor strength, sensory, reflex, coordination, and gait examination. Each of these components is worth 1 point. The checklist contains 10 items that assess the cranial nerve exam, 2 items that assess the reflex exam, and 1 item for each remaining category. These checklist items were weighted to generate the 1 point per component for a total possible 6 points [6].

The previous study, Standardized patient outcomes trial(SPOT) in neurology, showed that the average score for the NPE was 3.5 points, while the standard deviation was 1.1 [6]. A total of 34 study participants were required to obtain a difference of 1.3 or more at the significance level of 0.05, power of 0.9, and a 10% dropout rate. The study’s statistical analyses were performed using SAS software version 9.4 (SAS Institute, Cary, NC, USA). Demographics were compared using a *t*–test and Chi-squared test. The NPE scores, student satisfaction, and the realness of the CPX scenario were compared using a *t*-test and defined as significant when the *p*-value was 0.05 or less.

## Results

### Demographics

The study was conducted on 95 of 111 medical students completing an emergency medicine clerkship; 39 students used VRNET to examine SPs with dizziness and 56 students examined SPs without VRNET through conventional methods (Fig. 4). The mean age of the study subjects was 25.08 years (± 1.88), and the sex distribution was as follows: 68 males (71.58%) and 27 females (28.42%). The mean age of the SP group was 25.2 years (± 1.8), and the sex distribution was as follows: 37 males (66.1%) and 19 females (33.9%). The mean age of the SP with VRNET group was 24.9 years (± 2.0), and the sex distribution was as follows: 31 males (79.5%) and 8 females (20.5%). (Table 1).

**Table 1:** Demographics of the two groups

	SP (***N*** = 56)	SP with VRNET (***N*** = 39)	***p***-value
**Age(years)**^a^	25.2 ± 1.8	24.9 ± 2.0	0.425
**Male (%)**	37 (66.1%)	31 (79.5%)	0.146

^a^Data are expressed mean ± standard deviation

### Performance

#### The realness and satisfaction of VRNET

There were no statistical differences found in realness (SP group 4.27 ± 0.75, SP with VRNET group 4.28 ± 0.56, *p* = 0.92) and satisfaction (SP group 4.23 ± 0.71, SP with VRNET group 4.21 ± 0.66, *p* = 0.839) of students.

#### NPE score according to teaching methods

There was a statistically significant difference in the NPE score (*p* = 0.043); the SP + VRNET group had higher NPE scores (3.81 ± 0.92) than the SP group (3.40 ± 1.01). (Table 2).

**Table 2:** Realness, satisfaction, and NPE score of students according to teaching methods

	Method	Number	Score	***p***-value
**Realness**	SP	56	4.27 ± 0.75	0.92
	SP with VRNET	39	4.28 ± 0.56	
**Satisfaction**	SP	56	4.23 ± 0.71	0.849
	SP with VRNET	39	4.21 ± 0.66	
**NPE score**	SP	56	3.40 ± 1.01	0.043
	SP with VRNET	39	3.81 ± 0.92	

## Discussion

We developed VRNET and applied it to CPX education for the senior medical students in the study. This study evaluated the usefulness of VRNET by conducting a prospective randomized single-blind trial. This study showed that teaching neurological examination with VRNET produced similar levels of realness and satisfaction and higher NPE scores compared to those obtained after SP-based education.

VRNET offers many improvements; it offers the function of expressing involuntary neurologic symptoms, which SPs cannot express, and it also allows users to adjust the severity of the symptoms. These functionalities allow users to adjust each case to be similar to that of an actual patient. In addition, users can set various neurological abnormalities; this allows for the implementation of varied clinical cases. In the previous study, SPOT in neurology, which evaluated interns’ ability to examine patients who experienced consciousness-related changes, the scores were measured by observations of the history and physical examinations of the SP nurses and the simulator, which only implemented heart sounds, breath sounds, pulse rates, and blood pressure [10]. This study had some limitations. It assumed that the simulators, which could not express abnormal neurologic symptoms, could represent patients with neurologic abnormalities. In another study, which evaluated first-year residents in order to assess their physical examination ability, patients with abnormal findings were recruited; however, this study had the limitation that students were only trained to deal with abnormal patient findings [11].

One of VRNET’s limitations is that it offers a limited capability to express involuntary neurologic symptoms—that is, this expression functionality is limited to the cranial nerve function in the face area. However, the student can obtain realistic experience with paralysis and paresthesia of the limbs by utilizing the SP; therefore, when VRNET and SP present at the same time, an educational environment similar to that with an actual patient can be created. However, if the student performs a neurological examination involving cranial nerve abnormalities with VRNET and continues the rest of this examination using an SP, then the sense of realness may be broken. In the future, VRNETs should evolve and gain the capability to present all neurological abnormalities, thus eliminating the need for SPs.

With the widespread use and popularization of VR technology, numerous attempts have been made to teach medical students skills that are uncommon or those that cannot be learned by using actual patients. Shao et al. reported that knowledge, analysis, surgical technique, and comprehensive evaluation increased when VR technology was utilized in a neurosurgical class about skull base tumors [12]. Blumstein et al. reported that the utilization of VR technology-based teaching methods for intramedullary nailing related to tibia shaft fracture surgery increased aggregated global assessment scores compared to conventional teaching methods [13]. Students who had a VR experience of temporal bone mastoidectomy surgery showed higher satisfaction and otolaryngology interest [14]. Use of VR ophthalmoscopy for teaching ophthalmologists improved their understanding of testing processes and their identification of abnormal findings [15]. Anatomy classes also utilize VR technology in a variety of ways [12, 16].

In some cases, the clinical situation or the class situation itself—not just education on one specific skill—may be implemented using VR. Some studies have shown that students who experienced a VR situation in which cancer patients complained of nausea and vomiting showed good comfortability, concentration, and preference toward novel teaching [17]. When a 3D virtual classroom was implemented to lecture learners about abdominal imaging findings, the effect was not inferior compared to conventional lectures [18]. Students who experienced radiology lectures in Medical Master Island, which was created using the commercialized VR program, Second Life, expressed higher learning satisfaction [19]. When 3D VR was utilized to teach pharmacology mechanisms to nursing students, they obtained higher post-training test scores, as compared to scores obtained after 2D training [20]. VR has also been used for teaching appropriate patient posture when taking radiographs [21].

VR training offers many advantages over traditional lectures or SPs because it can provide an immersive learning environment and can be repeated; furthermore, it can overcome time and place barriers. However, there is little evidence that VR technology can completely replace current medical education curriculums, which depend on real patients to supplement medical knowledge. In this study, students evaluated the realness and satisfaction offered by VRNET as similar to that experienced when using an SP; however, because VRNET was only used for neurological examinations in this study, a patient history should be taken when using an SP. Furthermore, the neurological examination was continued using VRNET, and the interpretation and plan were explained to the SP again. This is because VRNET does not offer a conversation function. Given that previous studies have pegged the accuracy of VR patient responses at 79 to 86% [22], it is not yet possible to replace real-world patient care experiences with VR alone. In addition, most VR-related research studies have reported that previously unused and novel teaching techniques and knowledge demonstrate good educational effects. This may explain why novices tend to assign high scores to VR education; they may not have experiences with “real clinical experience.” As student participants of this study were the fourth years, they had already interacted with “real” patients, and VRNET, therefore, did not provide them with a higher sense of “realness” or “satisfaction” compared to conventional methods with SPs.

In medical education and simulation, costs are often difficult to assess and, when attempts are made, are frequently under-reported [23]. Developing a VR program, purchasing equipment such as high-performance hard-wares, effective graphics cards, accurate tracking systems, high-resolution monitors, etc., and allocating space to use VR technology for medical education are, of course, more expensive than traditional education. The higher the realism, the higher the cost [24]. Other studies have also considered this cost-effectiveness [25, 26]. However, if the recent decreasing VR costs and labor costs for education are considered as a whole, education using VR technology can be cost-effective. In a study comparing rehabilitation programs for stroke patients, it cost less when providing rehabilitation treatment at home with a VR program than when receiving rehabilitation treatment at a conventional hospital [27]. In another study, offering VR cognitive behavioral therapy to patients with paranoid delusions was an economically viable approach toward cost-effectively improving patients’ health [28].

We think that VR can be advantageous in terms of cost-effectiveness as the labor cost is much expensive and more 1:1 personal training is required because the VR education can be repeated. As well as financial savings, such technologies can save faculty time and space. Some VR setups do not need a faculty member to present on the education site. VR can deliver the clinical scenario in a small space (2 × 2 m) with under 5 min of setup [5].

### Limitations

We conducted a randomized control study to reduce the differences between the control and experimental groups; however, we were unable to measure students’ ability with regard to neurological examination skills within each group. Second, the NPE scores for the objective usefulness of VRNET as a teaching tool were statistically significant; however, it should be noted that it is unclear whether the difference of 0.4 points had any actual meaning in this context. Furthermore, we could not confirm the validity and reliability of our questionnaire and NPE score although it was designed by a neurologist and educators from the Margaret and Ian Smith Clinical Skills Center in previous studies [6]. Third, the main advantage of VRNET is that it allows students to directly catch and assess involuntary neurologic symptoms, which SPs cannot express; however, this study did not evaluate whether the students recognized abnormal neurological findings and evaluated this as an abnormal finding. Last, this study’s results cannot be directly applied to actual clinical situations because such situations differ from controlled CPX environments.

## Conclusion

VRNET is useful in teaching senior (graduating) medical students with SP with a neurologic problem.

## Supplementary Information

**Additional file 1.** Student Checklist.

## Data Availability

Anonymous data of the results of the evaluations could ask for the authors.

## Abbreviations

VR: Virtual reality
VRNET: Virtual reality-based neurological examination teaching tool
SP: Standardized patient
SPs: Standardized patients
NPE: Neurologic physical exam
CPX: Clinical performance examination
UI: User interface
IRB: Institutional review board
SPOT: Standardized patient outcomes trial
