# Supplementary material for: Virtual reality-based neurological examination teaching tool(VRNET) versus standardized patient in teaching neurological examinations for the medical students: a randomized, single-blind study
Source: BMC Med Educ. 2021 Sep 15;21:493. doi: 10.1186/s12909-021-02920-4 (PMC8444400; doi:10.1186/s12909-021-02920-4)
Supplement: Supplementary file 1 — Additional file 1. Student Checklist. [file 12909_2021_2920_MOESM1_ESM.docx]

**Appendix 1. Student Checklist**

Age _______ years

Gender (Male/Female)

**Please score on a 1-5 scale.**

This Clinical Performance Examination (CPX) training:

**Realness:**

To what extent did the training you received through SP with (or without) VRNET feel realistic?

Please check on scale of 1 (very unrealistic) to 5 (very realistic).

1 (Very unrealistic) ----- 2 ----- 3 ----- 4 ----- 5 (Very realistic)

**Satisfaction:**

To what extent were you satisfied with the training provided through SP with (or without) VRNET as an educational content?

Please check on scale of 1 (very dissatisfied) to 5 (very satisfied).

1 (Very dissatisfied) ----- 2 ----- 3 ----- 4 ----- 5 (Very satisfied)
